# Access to dental care and blood pressure profiles in adults with high socioeconomic status

**DOI:** 10.1002/JPER.21-0439

**Published:** 2021-12-21

**Authors:** Rita Del Pinto, Annalisa Monaco, Eleonora Ortu, Marta Czesnikiewicz‐Guzik, Eva Muñoz Aguilera, Mario Giannoni, Francesco D'Aiuto, Tomasz J. Guzik, Claudio Ferri, Davide Pietropaoli

**Affiliations:** ^1^ Unit of Internal Medicine and Nephrology – Center for Hypertension and Cardiovascular Prevention – San Salvatore Hospital, Department of Life, Health and Environmental Sciences University of L'Aquila L'Aquila Italy; ^2^ Oral DISeases and SYstemic interactions study group (ODISSY group) L'Aquila Italy; ^3^ Center for Oral Diseases, Prevention and Translational Research – San Salvatore Hospital, Department of Life, Health and Environmental Sciences Dental Clinic – University of L'Aquila L'Aquila Italy; ^4^ Department of Periodontology and Oral Sciences Research Group University of Glasgow Dental School Glasgow UK; ^5^ Department of Dental Prophylaxis and Experimental Dentistry, Jagiellonian University Collegium Medicum Krakow Poland; ^6^ Periodontology Unit, UCL Eastman Dental Institute and Hospital University College London London UK; ^7^ Institute of Cardiovascular and Medical Sciences University of Glasgow Glasgow UK; ^8^ Department of Internal and Agricultural Medicine, Collegium Medicum Jagiellonian University Krakow Poland

**Keywords:** blood pressure, dental care, inflammation, machine learning, oral health, socioeconomic factors

## Abstract

**Background:**

Reduced access to dental care may increase cardiovascular risk; however, socioeconomic factors are believed to confound the associations. We hypothesized that the relation persists despite economic wellness and high education, with reduced access to dental care affecting cardiovascular risk at least in part through its effect on blood pressure (BP), possibly mediated by systemic inflammation.

**Methods:**

We first assessed the sociodemographic and clinical characteristics related to last dental visit timing (≤ or >6 months; self‐reported) using national representative cross‐sectional data. Then, the association of last dental visit timing with clinic BP was selectively investigated in highly educated, high income participants, further matched for residual demographic and clinical confounders using propensity score matching (PSM). The mediating effect of systemic inflammation was formally tested. Machine learning was implemented to investigate the added value of dental visits in predicting high BP over the variables included in the Framingham Hypertension Risk Score among individuals without an established diagnosis of hypertension.

**Results:**

Of 27,725 participants included in the population analysis, 46% attended a dental visit ≤6 months. In the PSM cohort (*n* = 2350), last dental visit attendance >6 months was consistently associated with 2 mmHg higher systolic BP (*P* = 0.001) and with 23 to 35% higher odds of high/uncontrolled BP compared with attendance ≤6 months. Inflammation mildly mediated the association. Access to dental care improved the prediction of high BP by 2%.

**Conclusions:**

Dental care use impacts on BP profiles independent of socioeconomic confounders, possibly through systemic inflammation. Regular dental visits may contribute to preventive medicine.

## 1 INTRODUCTION

Hypertension is a major cardiovascular risk factor and a leading contributor to morbidity and mortality related to cardiovascular diseases (CVD). The latter represent the most common non‐communicable diseases globally and a leading cause of death worldwide,^1^ causing 45% of total mortality in Europe in 2017.^2^ Direct and indirect health costs related to hypertension and CVDs are astounding, and reducing systolic blood pressure (BP) by only 1 mmHg was estimated to translate into a $100,000 annual saving per hypertensive individual.^3^ In this context, the Global Hearts Initiative has recently promoted a comprehensive approach for cardiovascular prevention that is based on integrated primary health care interventions.^4^

As an inflammatory disease of the mouth increasing systemic inflammation, periodontitis has been recently acknowledged as an emerging contributor to cardiovascular risk.^5^ Consistent with this, dental care use appears to carry implications for cardiovascular health.^6^, ^7^, ^8^ Attending a dental visit on a regular basis^9^ or within the previous year,^10^ in fact, was associated with reduced risk of stroke, and dental visits for professional cleaning with at least annual frequency were shown to reduce cardiovascular risk by 14%.^6^ In parallel, periodontitis is associated with 20% increased risk of high BP or uncontrolled hypertension,^11^ and bleeding gums with or without periodontitis are associated with further increase in the same risk by an additional 20%,^12^ with a possible mediating effect of inflammation.^13^, ^14^ Nevertheless, socioeconomic factors—especially income and education—are often believed to largely account for the associations,^15^, ^16^ and studies that control for these sources of confounding are lacking. There is also a lack of data that might provide a deeper insight into the issue of professional dental care fruition for integrated cardiovascular prevention.

We hypothesized that a missing piece in the associations between reduced access to dental care and increased cardiovascular risk could be attributable to the impact of neglected oral care on BP possibly mediated by inflammation. We tested our hypothesis in a setting where access to cures is not limited by economic capacity, nor by educational issues. Therefore, the aims of this study were to investigate the sociodemographic and clinical features related to access to dental care at the population level, and to explore the association of access to dental care patterns with BP and the possible mediating effect of inflammation in a subset of highly educated, high‐income individuals.

## 2 MATERIALS AND METHODS

### 2.1 Study population

The National Health and Nutrition Examination Survey (NHANES) is a population‐based program of studies following a complex, stratified, multistage, probability‐cluster design to select a nationally representative sample of the United States civilian, non‐institutionalized population. We selected NHANES cycles where dental visit attendance was investigated with a specific question (OHQ030: “*About how long has it been since you last visited a dentist? Include all types of dentists, such as, orthodontists, oral surgeons, and all other dental specialists, as well as dental hygienists*”). Seven survey waves from 1999‐2004 to 2011‐2018 were selected, and a total of 27,725 individuals with complete information on both last dental visit and BP assessments were included (see Supplementary Figure S1 in online *Journal of Periodontology*).

### 2.2 Classification of dental visits attendance

The timing of the last dental visit for each participant as reported by NHANES was dichotomized as attendance within or before the previous 6 months (≤ or >6 months).^17^

In terms of dental visits indications, four strata as collected by NHANES were identified: check‐up/exam/hygiene; called for checkup/exam/hygiene; something was wrong/bothering/hurting; treatment of condition discovered at check‐up/exam. For brevity, these strata are presented here as “scheduled appointment,” “recall visit,” “something wrong,” and “treatment needed,” respectively.

### 2.3 Markers of oral health status

Information on the total number of permanent teeth, missing teeth for any cause, dental implants, as well as on periodontal indices (clinical attachment loss, CAL; periodontal probing depth, PPD; bleeding on probing, BoP) was reported as collected by NHANES.^18^, ^19^

Given the inter‐waves variability of periodontal assessment methods (full‐mouth for NHANES waves from 2009 to 2014; partial‐mouth for the years from 1999 to 2004^19^, ^20^), a case definition of periodontitis was not applied, and periodontal indices (BoP, PPD, and CAL) are presented as continuous variables and used for descriptive purposes only.

### 2.4 Blood pressure measurement and classification

The average (mean ± standard deviation, SD) of three valid seated, consecutive brachial BP readings obtained by trained personnel at a single examination visit following a standard protocol was used in this study for systolic and diastolic BP values (mmHg).

BP was also modeled as a categorical variable using the thresholds for the definition of hypertension diagnosis and control according to the American College of Cardiology/American Heart Association (ACC/AHA) (130/80 mmHg)^21^ and the European Society of Cardiology/European Society of Hypertension (ESC/ESH) (140/90 mmHg).^22^ BP values greater than or equal to these cutoffs were regarded as hypertension in untreated patients, and uncontrolled hypertension among those taking prescribed BP medications, and were labeled as “high/uncontrolled BP.” Otherwise, they were classified as “normal/controlled BP.”

### 2.5 Classification of covariates

Sociodemographic characteristics of interest included: age; sex; race/ethnicity; education; income; health insurance status; and dental coverage. Clinical characteristics of interest included serum inflammatory markers (C‐reactive protein [CRP]; high‐sensitivity CRP [hsCRP]; white blood cells [WBC]); glycohemoglobin A1c (HbA1c); low‐density lipoprotein cholesterol (LDL‐c); triglycerides; and body mass index (BMI). Information on the last medical visit, as well as the available data on concomitant diseases (self‐reported history of angina, congestive heart failure [CHF], coronary artery disease [CAD], heart attack, stroke, asthma, emphysema, chronic bronchitis, liver diseases, arthritis, cancer, as collected by NHANES), and smoking status were also collected.

### 2.6 Statistical analysis

The goals of this study were: (1) to assess the sociodemographic and clinical features related to access to dental care in a large, real‐world setting, with focus on BP and (2) to test the association of access to dental care, as assessed by the timing of last dental visit, with BP in highly educated, high‐income individuals (i.e., at least college graduates with >350% of the federal poverty level, FPL).^23^

Because the timing of dental visits might have reflected participants’ health awareness, access to healthcare, and/or global health status, we controlled for possible additional confounders in the association analysis, besides socioeconomic factors, by applying propensity score matching (PSM) to the subgroup of highly educated, high‐income NHANES participants. PSM is a technique used in observational studies to aid in the evaluation of cause–effect hypotheses and to reduce bias in the effect estimates by ensuring balance in the observed variables between groups.^24^, ^25^ Specifically, 1:1 nearest‐neighbor matching was performed for age (smoothed function), sex, race/ethnicity, BMI, glycolipid profile, comorbidities, and smoking habits in the selected subgroup using logistic generalized additive model (GAM), achieving balance in terms of the mentioned confounders using a specific R package.^26^ Univariate balance summary statistics and visual depictions of distribution for each covariate were performed using the same library.^26^ Variables multicollinearity were tested prior to applying PSM using a bootstrap stepwise algorithm.^27^ BP means and univariate odds ratios (ORs) for high/uncontrolled BP according to the timing of last dental visit (≤ or >6 months) were then obtained from logistic generalized linear model (GLM), where BP was the dependent variable. Further adjustment was made for insurance status (bivariate model), for its possible impact on BP management and treatment. Stratification was made based on the main reason for dental visit, the identification of treatment needs (periodontal treatment, dental hygiene, dental restoration), and the presence of dental implants. A subgroup analysis excluding those not taking the prescribed antihypertensive medications was also performed.

Formal mediation analysis was performed to test if the effect of dental visit reason (exposure) on the likelihood of high/uncontrolled BP (outcome) was mediated by hsCRP and WBC (mediators).^28^ Dental visit reason, and not their timing, was used as the exposure in the hypothesis that it would be more sensitive than timing in the assessment of a mediation effect of inflammation on BP, given the variety of conditions that prompted the dental visit and, consequently, the possibly different burden of local inflammation.

Finally, a highly effective machine learning classification technique, implemented by random forest using 10‐fold cross‐validation, was used to assess variables importance in predicting high BP (≥130/80 and ≥140/90 mmHg). Machine learning was trained in the PSM subset after exclusion of patients with an established diagnosis of hypertension (*N* = 639). Then, performance of access to dental care in predicting high BP was assessed by calculating its capacity to provide additional information compared with the variables used in the Framingham Hypertension Risk Score (age; sex; BMI; cigarette smoking).^29^ To this aim, two models were instructed that differed only for the inclusion of dental care access. Hypertension family history was unavailable from NHANES and was therefore not included.

Complex multistage weighted probability samples were used for descriptive statistics for representativeness of the reference population,^30^ whereas unweighted estimates were adopted for association analyses on the specific PSM subset.^31^ Population based estimates were evaluated with unpaired t tests for continuous variables and χ^2^ tests for categorical variables, whereas Wilcoxon test was used for comparing means in the PSM subset.^32^ Covariates had <3% of missing data, and no imputations were applied.^33^ Statistical analyses were performed using R (v 4.0.2).

## 3 RESULTS

### 3.1 Population‐based descriptive analysis

Of the 27,725 included participants, comparable to 1.17 billion people, 46% reported a dental visit within the previous 6 months (see Supplementary Table S1 in online *Journal of Periodontology*). They had lower BP, were less likely diagnosed with hypertension, and more likely reported taking the prescribed antihypertensive medications than the counterpart. They tended to be middle aged, highly educated, high‐income, non‐smoker, non‐Hispanic White women, with healthier periodontium, lower levels of serum inflammatory markers, lower BMI, more favorable glycolipid profile, and were more likely to have health insurance and dental service coverage. They had less comorbidities, but more prevalent cancer. Their main reason for dental visit was checkup, exam, or dental hygiene. Only a minority reported a medical visit in the previous 6 months.

Conversely, participants who reported not having attended a dental visit in the previous 6 months were younger, non‐White, middle income men with more comorbidities, neglected oral care, worse glycolipid profile, higher levels of serum inflammatory markers, and higher BP. They had more prevalent cardiovascular and respiratory diseases. Their main reason for accessing dental care were symptoms, and they more often needed further treatment. Interestingly, they more likely attended a dental visit than a medical visit in the previous year.

Further investigation on the timing of medical and dental visits revealed that 26.5% of individuals attended a dental office, whereas only 3.1% attended a general healthcare visit within the previous 6 months (*P* < 0.001). These findings were confirmed throughout age categories (see Supplementary Table S2 in online *Journal of Periodontology*).

Socioeconomic and ethnic/racial features (e.g., income, education, minorities) consistently impacted on dental visits attendance across the examined NHANES cycles (see Supplementary Table S3 in online *Journal of Periodontology*).

### 3.2 PSM cohort for association analysis

The PSM cohort comprised 2350 highly educated, high‐income participants equally stratified in two groups (*N* = 1175 per group) based on last dental visit attendance (≤ or >6 months) (Table 1). Participants who attended a dental visit ≤6 months had less PPD and BoP, more permanent teeth and dental implants, lower WBC, lower systolic BP, and were more likely to achieve BP goals than the counterpart. They more likely had healthcare insurance.

**TABLE 1:** Demographic and clinical characteristics of highly educated, high‐income NHANES participants included in the PSM cohort (*n* = 2350; 100% college graduate or above and PIR >350%), equally stratified according to the timing of the last dental visit (≤ or >6 months)

Variables	Strata	≤6 Months	>6 Months	*P*‐value
*N*		1175	1175	
Female (%)		551 (46.9)	537 (45.7)	0.591
Age (%)	<45	553 (47.1)	546 (46.5)	0.833
	45‐65	466 (39.7)	463 (39.4)	
	>65	156 (13.3)	166 (14.1)	
Race (%)	Mexican American	59 (5.0)	67 (5.7)	0.825
	Other Hispanic	44 (3.7)	53 (4.5)	
	NH White	584 (49.7)	577 (49.1)	
	NH Black	207 (17.6)	205 (17.4)	
	Other race	281 (23.9)	273 (23.2)	
Ethnicity (%)	Hispanic	103 (8.8)	120 (10.2)	0.260
BMI (mean [SD])		28.22 (6.01)	28.40 (6.18)	0.474
BMI categories (%)	Underweight	11 (0.9)	16 (1.4)	0.791
	Normal	367 (31.2)	365 (31.1)	
	Overweight	405 (34.5)	397 (33.8)	
	Obese	392 (33.4)	397 (33.8)	
WBC (mean [SD])		6.59 (1.78)	6.88 (2.67)	0.002
Lymphocytes (mean [SD])		1.99 (0.62)	2.13 (1.92)	0.015
Neutrophils (mean [SD])		3.85 (1.45)	3.97 (1.48)	0.051
LDL (mean [SD])		117.07 (35.38)	118.75 (34.55)	0.471
Triglycerides (mean [SD])		118.89 (78.85)	137.56 (194.93)	0.053
HbA1c (mean [SD])		5.57 (0.77)	5.61 (0.97)	0.205
Self‐reported diabetes (%)		105 (8.9)	93 (7.9)	0.383
Insulin therapy (%)		31 (2.8)	24 (2.1)	0.367
Comorbidities (%)		91 (47.6)	80 (51.9)	0.492
N. of comorbidities (mean [SD])		0.81 (1.21)	0.93 (1.24)	0.356
Asthma (%)		150 (12.8)	140 (11.9)	0.583
CHF (%)		12 (1.0)	15 (1.3)	0.699
CAD (%)		38 (3.2)	32 (2.7)	0.544
Angina (%)		24 (2.0)	14 (1.2)	0.144
Heart attack (%)		27 (2.3)	26 (2.2)	1.000
Stroke (%)		19 (1.6)	17 (1.4)	0.871
Emphysema (%)		5 (0.4)	3 (0.3)	0.722
Chronic bronchitis (%)		44 (3.7)	31 (2.6)	0.161
Liver diseases (%)		31 (2.6)	41 (3.5)	0.283
Arthritis (%)	Osteoarthritis	59 (30.1)	47 (29.9)	0.317
	Psoriatic arthritis	7 (3.6)	11 (7.0)	
	Rheumatoid arthritis	110 (56.1)	89 (56.7)	
	Other	20 (10.2)	10 (6.4)	
Cancer (%)		105 (8.9)	115 (9.8)	0.524
CRP (mean [SD])		0.35 (0.57)	0.34 (0.52)	0.951
hs‐CRP (mean [SD])		3.14 (5.41)	3.21 (5.57)	0.873
HT diagnosis (%)		343 (29.2)	348 (29.7)	0.835
HT prescriptions (%)		291 (84.8)	280 (80.7)	0.180
Now taking HT drugs (%)		259 (89.0)	242 (86.4)	0.418
Controlled HT (ACC/AHA guidelines)	<130/80 mmHg	746 (63.5)	691 (58.8)	0.022
Controlled HT (ESC/ESH guidelines)	<140/90 mmHg	1010 (86.0)	965 (82.1)	0.013
SBP (mean [SD])		121.23 (16.38)	123.56 (17.41)	0.001
DBP (mean [SD])		72.53 (10.30)	73.18 (11.40)	0.144
CAL (mean [SD])		1.07 (0.66)	1.11 (0.74)	0.319
PPD (mean [SD])		1.09 (0.40)	1.17 (0.46)	0.001
BoP (mean [SD])		4.26 (7.22)	6.86 (10.30)	0.002
Missing teeth (mean [SD])		6.42 (6.47)	7.31 (7.94)	0.003
Dental implants (%)		75 (6.6)	36 (3.2)	<0.001
Smoking (%)		353 (30.0)	366 (31.1)	0.591
Health insurance (%)		1167 (99.3)	1111 (94.6)	<0.001
Dental coverage (%)		275 (73.7)	241 (68.3)	0.124
Last medical visit (%)	<6 Months	0 (0.0)	2 (2.1)	0.714
	<1 Year	1 (2.7)	5 (5.2)	
	<3 Years	27 (73.0)	61 (62.9)	
	≥3 Years	9 (24.3)	28 (28.9)	
	Never	0 (0.0)	1 (1.0)	
Last dental visit (%)	≤6 Months	1175 (100.0)	0 (0.0)	<0.001
	<1 Year	0 (0.0)	517 (44.0)	
	<2 Years	0 (0.0)	295 (25.1)	
	<3 Years	0 (0.0)	141 (12.0)	
	<5 Years	0 (0.0)	107 (9.1)	
	≥5 Years	0 (0.0)	115 (9.8)	
Dental visit reasons (%)	Called for check‐up/exam/clean	94 (8.0)	68 (5.8)	<0.001
	Check‐up/exam/clean	854 (72.7)	776 (66.0)	
	Something was wrong/bothering/hurting	123 (10.5)	216 (18.4)	
	Treatment of condition discovered at check‐up/exam	94 (8.0)	93 (7.9)	
Recommendation for cure (%)	Dentist within 2 weeks	5 (0.4)	29 (2.6)	<0.001
	Dentist earliest convenience	277 (24.4)	458 (40.8)	
	Continue regular routine care	855 (75.2)	635 (56.6)	
NHANES cycles (%)	1999‐2000	117 (10.0)	86 (7.3)	0.045
	2001‐2002	139 (11.8)	158 (13.4)	
	2003‐2004	123 (10.5)	140 (11.9)	
	2011‐2012	217 (18.5)	202 (17.2)	
	2013‐2014	227 (19.3)	209 (17.8)	
	2015‐2016	179 (15.2)	167 (14.2)	
	2017‐2018	173 (14.7)	213 (18.1)	

ACC, American College of Cardiology; AHA, American Heart Association; BMI, Body Mass Index; BoP, bleeding on probing; CAD, coronary artery disease; CAL, clinical attachment loss; CHF, congestive heart failure; CRP, C‐reactive protein; DBP, diastolic blood pressure; ESC, European Society of Cardiology; ESH, European Society of Hypertension; HbA1C, glycohemoglobin; HS‐CRP, high sensitive C‐reactive protein; HT, hypertension; LDL, low‐density cholesterol; NH, non‐Hispanic; NHANES, National Health and Nutrition Examination Survey; PIR, poverty‐income ratio; PPD, periodontal probing depth; SBP, systolic blood pressure; SD, standard deviation; WBC, white blood cells.

Participants who attended a dental visit >6 months had 2.33 mmHg higher mean systolic BP (*P* = 0.001) (Table 1) and +22 to +33% the odds of high/uncontroled BP (OR 1.22, 95% CI 1.03 to 1.44, *P* = 0.020 and OR 1.33, 95% CI 1.07 to 1.66, *P* = 0.011 according to the US and the European guidelines, respectively) than the counterpart. After controling for healthcare insurance status, their OR (95% CI) of high/uncontroled BP was 1.23 (95% CI 1.04 to 1.46, *P* = 0.014) and 1.35 (95% CI 1.08 to 1.69, *P* = 0.009) according to the US and the European guidelines, respectively. Additional inclusion of survey years did not modify the results (data not shown).

**FIGURE 1:**
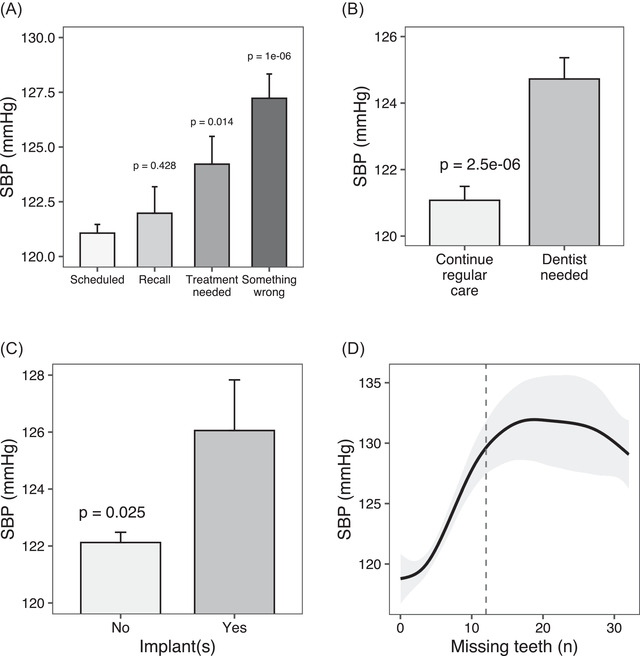
Systolic BP according to reasons for dental visit attendance (**panel A**), related recommendations (**panel B**), and selected clinical features (**panel** **s** **C and D**) in the PSM cohort. Panel A, B, C: mean systolic BP (SD) (mmHg) is reported. Panel A: scheduled visit is the reference. Panel D: cubic spline of the relationship between systolic BP (mmHg) and the number of missing teeth. The relation is linear up to 12 missing teeth (dotted line)

Mean systolic BP progressively increased from participants with a scheduled visit (*N* = 1630; 121.1 ± 16.0 mmHg), to those with a recall visit (*N* = 162; 122.0 ± 15.4 mmHg), treatment needs (*N* = 187; 124.2 ± 17.3 mmHg; +3.1 mmHg, *P* = 0.014), and something wrong (*N* = 339; 127.2 ± 20.4 mmHg; +6.1 mmHg, *P* < 0.001) (Figure 1A). In agreement with this, participants who did not need additional dental visits (N = 1490, 121.1 ± 16.1 mmHg) and those who had no dental implants (*N* = 2155, 122.1 ± 16.7 mmHg) had lower mean systolic BP than the counterparts (n.769, +3.4 mmHg, *P* < 0.001; and n.111, +4.0 mmHg, *P* = 0.025, respectively) (Figure 1B,C). The association of systolic BP with missing teeth was substantially linear up to the threshold of 12 missing teeth (Figure 1D).

Systolic BP progressively increased with clinical measures of gingival health (PPD, CAL), independent of access to dental care, and no difference in mean systolic BP was observed according to dental visits attendance given the same PPD (*P* = 0.397) or CAL (*P* = 0.309) (see Supplementary Figure S2 in online *Journal of Periodontology*). These findings were also confirmed in a restricted analysis on 766 participants from the 2011‐2014 NHANES waves, where full‐mouth, six‐sites periodontal assessment was performed (data not shown).

The subgroup analysis restricted to those taking the prescribed antihypertensive medications (*N* = 501) indicated a significant increase in the risk of BP ≥140/90 mmHg among individuals not attending the dental office in the previous 6 months (*N* = 259; OR 1.62, 95% CI: 1.11 to 2.36; *P* = 0.012). The addition of insurance status in the model did not modify the results. Their mean systolic BP tended to be 3.2 mmHg higher than the counterpart (134.3 ± 19.2 mmHg versus 131.1 ± 18.2 mmHg, *P* = 0.055). Their risk of BP ≥130/80 mmHg was not significantly increased (OR 1.26, 95% CI: 0.88 to 1.81, *P* = 0.199; and OR 1.25, 95% CI: 0.87 to 1.79, *P* = 0.230 in the univariate and the bivariate model, respectively).

**FIGURE 2:**
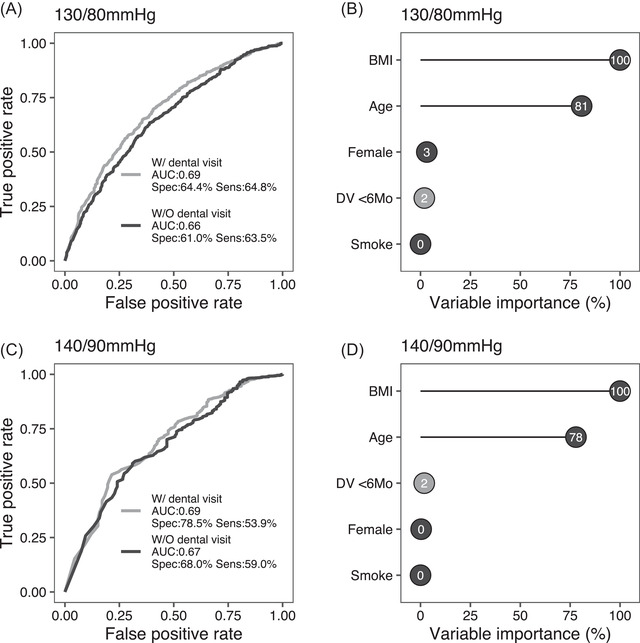
Improvement of the AUC‐ROC after the addition of last dental visit timing to the Framingham Hypertension Risk Score. **Panel** **s** **A and C**. The addition of the information on dental visit attendance >6 or ≤6 months significantly improved the AUC‐ROC for high BP (A: ≥130/80 mmHg; C: ≥140/90 mmHg) compared with the AUC‐ROC of the Framingham Hypertension Risk Score alone. **Panel** **s** **B and D**. Dental visit attendance >6 or ≤6 months ranked superior than cigarette smoking (B, D) and sex (D) in the variable importance analysis. AUC, area under the curve; DV, dental visit

Machine learning performed on participants without an established diagnosis of hypertension (*N* = 1657) indicated that information on access to dental care significantly improved the AUC‐ROC for high BP compared with the AUC‐ROC obtained without this variable (0.69 vs 0.66 to 0.67 depending on the BP threshold; Figure 2A,B). In particular, including dental visits attendance determined a gain in the model predictive power by 2% over the variables of the Framingham Hypertension Risk Score (Figure 2A,B), and improved the prediction of high BP more than cigarette smoking (Figure 2C,D).

**FIGURE 3:**
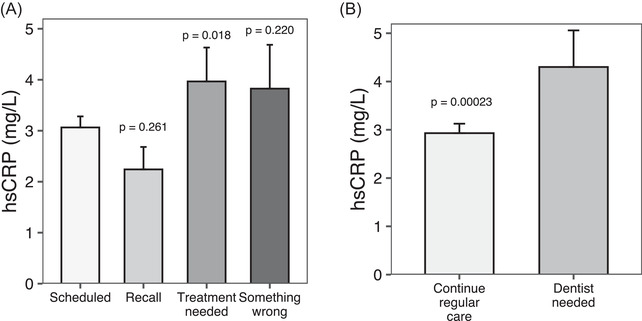
Serum hsCRP (SD) (mg/L) according to reasons for dental visit attendance (**panel A**) and related recommendations (**panel B**) in the PSM cohort. Panel A: scheduled visit is the reference

We then examined serum inflammatory markers (hsCRP, WBC) based on the reason for dental visits fruition and related recommendations. Mean serum hsCRP levels, available from the 2015‐2018 NHANES cycles (*N* = 719) [Citation error], were significantly higher among individuals who attended the dental office for some treatment need (*N* = 57; 4.0 ± 5.0 mg/L) compared with those having a scheduled appointment (*N* = 496, 3.1 ± 4.8 mg/L; mean difference 0.9 mg/L, *P* = 0.018) or a recall visit (*N* = 56, 2.2 ± 3.3 mg/L, mean difference 1.8 mg/L; *P* = 0.009) (Figure 3A). Similarly, mean serum hsCRP was higher among participants who were told they needed further assessments (*N* = 121; 4.3 ± 8.3 mg/L) compared with those who were told to continue with routine care (*N* = 577; 2.9 ± 4.7 mg/L; mean difference 1.4 mg/L, *P* < 0.001) (Figure 3B).

Individuals attending the dental office for a recall visit had lower total WBC than those who attended for treatment needs (*P* = 0.047) or for something wrong (*P* = 0.033).

**FIGURE 4:**
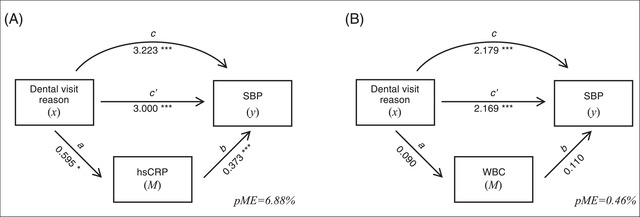
Formal mediation analysis relative to the mediation effect of serum hsCRP (**p** **anel A**) or WBC (**p** **anel B**) in the association between dental visit reason and systolic BP. Independent variable (*x*): reasons for dental visit attendance (scheduled appointment; recall visit; something wrong; treatment needed); dependent variable (*y*): systolic BP (mmHg); mediator (*M*): hsCRP (mg/dL) or WBC (count/uL). Direct effect of *x* on *y* (*c’*); effect of *x* on *M* (a); effect of *M* on *y* (b); total effect of *x* on *y* (c); proportion of the mediated effect (pEM). Independent categorical variable was converted into numeric as specified.^28^ ***P < 0.001; **P < 0.01; *P < 0.05

A progressive increase in systolic BP based on dental visits reason was also confirmed in the subset of participants with available hsCRP (scheduled visit: 121.5 ± 15.5 mmHg; recall visit: +2.6 mmHg, NS; treatment needed: +6.3 mmHg, *P* = 0.005; something wrong: +8.4 mmHg, *P* < 0.001). The formal mediation analysis indicated that hsCRP and WBC mediated 6.88% and 0.46% of the effect of dental visit reason on systolic BP, respectively (Figure 4A,B).

## 4 DISCUSSION

This population‐based analysis on NHANES adults indicates that access to dental care was associated with several demographic and clinical features, including BP profile and control, across all the examined survey campaigns. The association with BP persisted after controlling for the available confounders related to health awareness, access to healthcare, and global health status among propensity‐matched individuals in the top category of income and education. Not attending a dental office in the previous 6 months was associated with 2 mmHg higher systolic BP and with 23 to 35% higher odds of high/uncontrolled BP compared with attendance within 6 months. Having treatment needs or suffering from an acute oral issue was associated with worse systolic BP by 3 to 6 mmHg compared with professional oral care fruition for a scheduled appointment.

Although the exact mechanisms behind the reported associations remain to be elucidated, low‐grade systemic inflammation appears to contribute to the observed findings. The need for treatment, whether as the reason for the dental visit or as the related recommendation, was in fact associated with higher hsCRP by 0.9 to 1.4 mg/L and higher systolic BP compared with stable conditions. Mechanistically, nearly 7% of the effect of accessing dental care on BP was mediated by hsCRP. This is in line with the existing evidence of neglected oral care as a source of systemic inflammation with cardiovascular impact,^8^, ^13^ and expands current knowledge on its effects on BP as the bridge to cardiovascular implications.^9^

Our descriptive analysis is in line with previous evidence indicating an association between socioeconomic inequalities and oral health status, whether self‐reported or clinically diagnosed.^15^, ^34^ Specifically, it has been demonstrated that the socioeconomic position is negatively associated with oral health and clinically diagnosed dental disease.^15^, ^35^ Our propensity‐score matched analysis advances the available knowledge by showing an association between dental visits and BP despite economic wellness and high education.

Intriguingly, we observed that individuals with at least one dental implant had higher systolic BP compared with those without. This is an interesting observation, because recent findings support the hypothesis that titanium particles from implants might drive low‐grade inflammation.^36^ Specifically, histology shows that peri‐implant sites harbor more neutrophils, larger proportions of CD19+ cells^37^ and higher levels of inflammatory cytokines^38^ than control sites, which might translate into a plausible systemic impact.^39^

Hypertension itself is considered a condition associated with low‐grade inflammation involving both innate and adaptive immunity.^40^, ^41^ Toll‐like receptor 4 and activated perivascular T cells were shown to trigger vascular inflammation in response to hypertensive stimuli, ultimately leading to arterial remodeling and impaired vasoreactivity.^40^, ^42^ There is evidence of a hypertension‐specific immune host response to periodontal bacteria,^43^ and that the oral‐gut microbiota is involved in BP regulation.^44^ Importantly, periodontal treatment and a healthy oral microbiota significantly modulate adaptive immunity.^14^, ^45^ In this setting, better access to dental care, also by means of timely dental visits, might reduce the systemic inflammatory burden by a multiplicity of mechanisms, spanning immunity modulation to dysbiosis reversal.

We also observed that higher values of PPD and CAL were associated with worse systolic BP independent of the timing of the last dental visit. Identifying individuals with poor periodontal health, either despite regular dental visits or in relation to neglected oral care, might therefore be relevant in cardiovascular prevention.

Our findings also suggest that people attended the dentist more often than the medical doctor. Thus, oral health specialists might play a crucial role in detecting chronic diseases, related risk factors, and the conditions possibly affecting their progression and control.^46^ Such a contribution to preventive medicine might lighten the socioeconomic burden attributable to CVD.

This study has some limitations. Periodontal examination was performed according to different protocols across NHANES campaigns, which should be considered when interpreting the relative findings. BoP, which is associated with a worse BP profile,^12^ was not widely available. Findings might not be widely generalizable to other settings with different health and dental care access policies. The Framingham Hypertension Risk Score was developed to detect hypertension incidence in predominantly White people, although found to perform well also among non‐White individuals,^47^ and information on family history of hypertension was unavailable. The impact of unmeasured residual confounders, including unmeasured cultural determinants, home oral hygiene, and other diseases affecting dental visits attendance or systemic inflammation, could not be examined.^48^

This study also has several strengths. It is the first to examine the impact of reduced access to dental care on BP profile and control after exclusion of relevant socioeconomic bias sources. Besides improving internal validity, PSM also allowed for causal inferences in the context of an observational, cross‐sectional study, adding to previous literature where socioeconomic confounders were not controled for. A subgroup analysis excluding untreated hypertensive individuals was performed. A formal mediation analysis was performed, supporting systemic inflammation as a link between oral health behavior and BP. The added value of dental visits timing for high BP prediction was tested by a highly effective machine learning technique. In addition, we provide the first evidence of impaired BP profile in individuals with dental implants. The population‐based analysis was conducted on a large, representative sample of the multiethnic US population in a large time span.

## 5 CONCLUSIONS

In conclusion, access to dental care assessed by the timing of dental visits is related to BP profiles, possibly through systemic inflammation. Thus, cardiovascular prevention strategies might benefit from a comprehensive approach that includes timely dental visits.^49^ Future clinical trials on hypertension, as well as the clinical practice, might benefit from systematically assessing information on oral health status and access to dental care.

## FINANCIAL DISCLOSURE

RDP, MG, AM, and DP received a proportion of funding from Colgate‐Palmolive Italy SRL within “Colgate Prize 2020”. EMA and FD work at UCLH/UCL which received a proportion of funding from the Department of Health's NIHR Biomedical Research Centre funding scheme.

## CONFLICT OF INTEREST

The authors declare no conflicts of interest.

## AUTHOR CONTRIBUTIONS

DP and RDP contributed equally to this paper. DP and RDP contributed to the conception and the design of the work, acquisition and data analysis. DP, RDP, FDA, EMA, CF, AM, EO, MG, MCG, and TG contributed to interpretation of data for the work. DP, RDP, EO, and EMA drafted the manuscript. AM, MG, FDA, MCG, TG, and CF critically revised the manuscript. All gave final approval and agreed to be accountable for all aspects of work ensuring integrity and accuracy.

## Supporting information

Supplementary information

Supplementary information

Supplementary information

Supplementary information

Supplementary information

## Data Availability

Data can be accessed/required to the Center for Disease Control and Prevention cdc.gov.
