# Supplementary figures and images for: Access to dental care and blood pressure profiles in adults with high socioeconomic status
Source: J Periodontol. 2021 Dec 21;93(7):1060–71. doi: 10.1002/JPER.21-0439 (PMC9542004; doi:10.1002/JPER.21-0439)

**Supplementary Figure 1.** Data reduction diagram

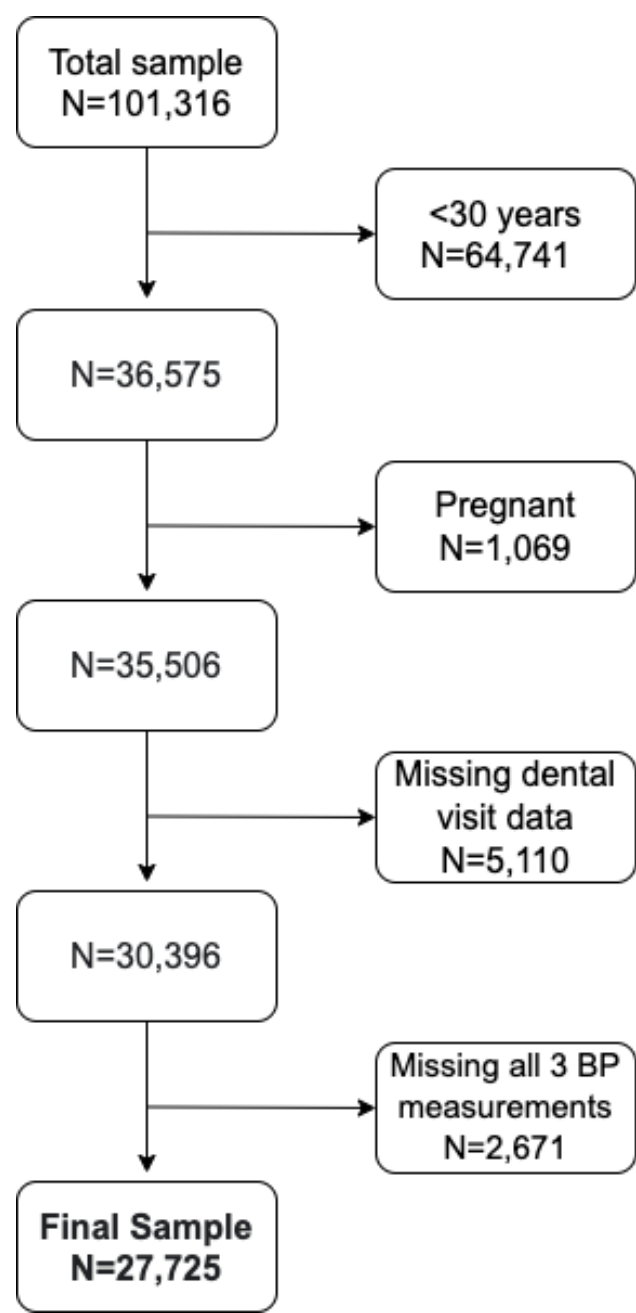

Supplement: Supplementary file 1 — Supplementary information [file JPER-93-1060-s005.pdf]
