# Supplementary material for: Access to dental care and blood pressure profiles in adults with high socioeconomic status
Source: J Periodontol. 2021 Dec 21;93(7):1060–71. doi: 10.1002/JPER.21-0439 (PMC9542004; doi:10.1002/JPER.21-0439)

Supplementary Figure 2. Cubic splines of the relationship between systolic BP (mmHg) and PPD (panel A) and CAL (panel B), stratified according to the timing of the last dental visit (≤ or >6 months), in the PSM cohort. The grey ribbons indicate the standard error (SE).

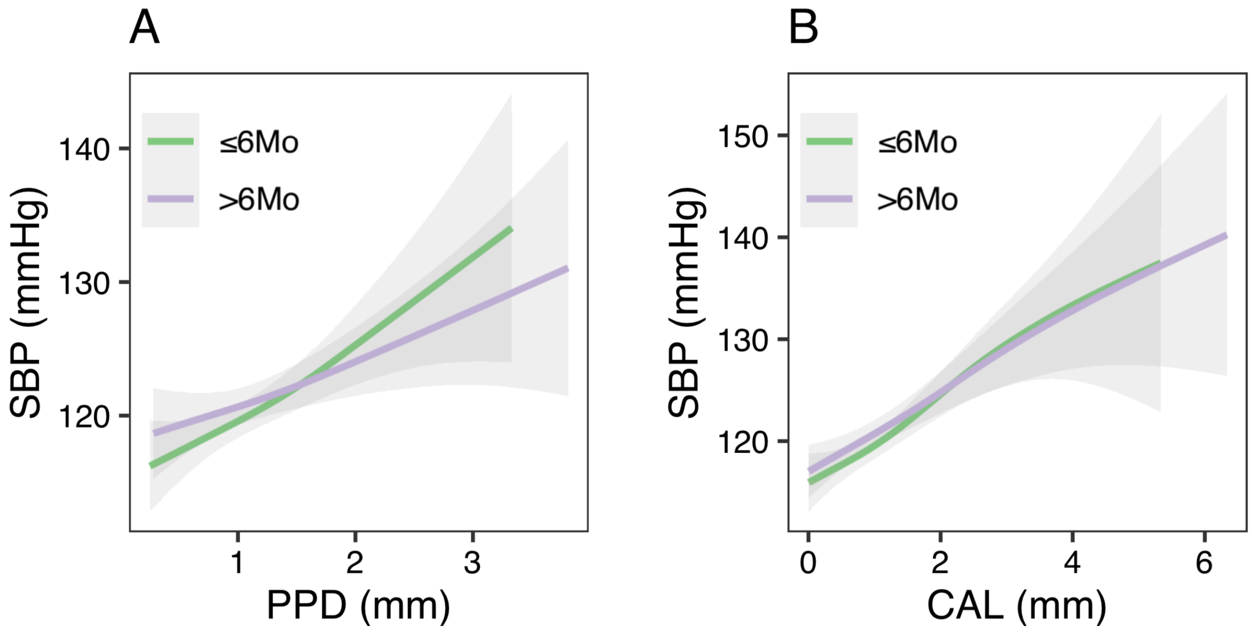

Supplement: Supplementary file 2 — Supplementary information [file JPER-93-1060-s004.pdf]
