# Supplementary material for: Access to dental care and blood pressure profiles in adults with high socioeconomic status
Source: J Periodontol. 2021 Dec 21;93(7):1060–71. doi: 10.1002/JPER.21-0439 (PMC9542004; doi:10.1002/JPER.21-0439)
Supplement: Supplementary file 3 — Supplementary information [file JPER-93-1060-s003.pdf]

Access to dental care and blood pressure profiles in adults with high socioeconomic status

Rita Del Pinto, Annalisa Monaco, Eleonora Ortu, Marta Czesnikiewicz-Guzik, Eva Muñoz Aguilera, Mario Giannoni, Francesco D'Aiuto, Tomasz J. Guzik, Claudio Ferri, Davide Pietropaoli

Supplementary material

Supplementary Table 1. Demographic and clinical characteristics of the 27,725 NHANES participants included in the population-based analysis (1,17 billion), stratified according to the timing of the last dental visit (≤ or >6 months).

Variables	Strata	Overall	≤6 Months	>6 Months	p-value
n		1168138636	542154328	625984308	
Female (%)		599299475 (51)	291069421 (54)	308230054 (49)	<0.001
Age (%)	<45	432705437 (37)	177297215 (33)	255408222 (41)	<0.001
	45-65	491935723 (42)	242027101 (45)	249908621 (40)	
	>65	243497477 (21)	122830013 (23)	120667464 (19)	
Race (%)	Mexican American	80884260 (7)	22948443 (4)	57935817 (9)	<0.001
	Other Hispanic	65787839 (6)	23428971 (4)	42358867 (7)	
	NH white	817487586 (70)	420938716 (78)	396548870 (63)	
	NH black	123265802 (11)	39748346 (7)	83517456 (13)	
	Other race	80713149 (7)	35089852 (6)	45623297 (7)	
Ethnicity (%)	Hispanic	146672099 (13)	46377414 (9)	100294684 (16)	<0.001

Education (%)	9-11th grade	127207690 (11)	33846972 (6)	93360718 (15)	<0.001
	College graduate or above	346396580 (30)	222578167 (41)	123818413 (20)	
	High school graduate/GED	274630375 (24)	106889540 (20)	167740835 (27)	
	Less than 9th grade	73663587 (6)	16563622 (3)	57099965 (9)	
	Some college/AA degree	345257603 (30)	162194752 (30)	183062851 (29)	
PIR (%)	<130%	212187816 (20)	54135686 (11)	158052130 (28)	<0.001
	130%-350%	379450663 (35)	147456609 (29)	231994055 (40)	
	>350%	486519245 (45)	303239108 (60)	183280138 (32)	
BMI (mean (SD))		29.1 (6.6)	28.6 (6.2)	29.6 (6.9)	<0.001
BMI categories (%)	Underweight	14221860 (1)	5822079 (1)	8399780 (1)	<0.001
	Normal	303673506 (26)	150729816 (28)	152943690 (25)	
	Overweight	399899175 (35)	197631849 (37)	202267326 (33)	
	Obese	434187908 (38)	182253335 (34)	251934573 (41)	
WBC (mean (SD))		7.2 (2.7)	7.0 (2.3)	7.4 (3.0)	<0.001
Lymphocytes (mean (SD))		2.1 (1.7)	2.0 (1.2)	2.2 (2.0)	<0.001
Neutrophils (mean (SD))		4.3 (1.7)	4.1 (1.6)	4.4 (1.7)	<0.001

LDL (mean (SD))		119.4 (35.6)	117.5 (34.6)	121.1 (36.4)	0.001
Triglycerides (mean (SD))		140.2 (129.7)	136.0 (113.1)	144.0 (142.8)	0.015
HbA1c (mean (SD))		5.7 (1.0)	5.6 (0.8)	5.7 (1.1)	<0.001
Self-reported diabetes (%)		128181850 (11)	50949211 (9)	77232640 (12)	<0.001
Insulin therapy (%)		34119855 (3)	12372980 (2)	21746875 (4)	<0.001
Comorbidities (%)		139431752 (58)	67222712 (55)	72209040 (60)	0,029
N. of comorbidities (mean (SD))		1.1 (1.4)	1.0 (1.2)	1.2 (1.5)	<0.001
Asthma (%)		153246021 (13)	69292322 (13)	83953699 (13)	0.252
CHF (%)		34371771 (3)	11406558 (2)	22965213 (4)	<0.001
CAD (%)		50995107 (4)	23527060 (4)	27468047 (4)	0.854
Angina (%)		36284353 (3)	14497714 (3)	21786639 (3)	0.001
Heart attack (%)		48776415 (4)	18758583 (3)	30017831 (5)	<0.001
Stroke (%)		38642667 (3)	13372820 (2)	25269847 (4)	<0.001
Emphysema (%)		25239201 (2)	8397839 (2)	16841362 (3)	<0.001
Chronic bronchitis (%)		79588350 (7)	31161834 (6)	48426515 (8)	<0.001
Liver disease (%)		50741679 (4)	22603846 (4)	28137833 (5)	0.362
Arthritis (%)	Osteoarthritis	73073620 (29)	32539098 (26)	40534522 (32)	<0.001
	Psoriatic arthritis	14147031 (6)	5840432 (5)	8306598 (7)	

	Rheumatoid arthritis	141336345 (56)	75851516 (60)	65484828 (52)	
	Other	22484431 (9)	11235150 (9)	11249282 (9)	
Cancer (%)		138385554 (12)	75438210 (14)	62947344 (10)	<0.001
CRP (mean (SD))		0.4 (0.8)	0.4 (0.7)	0.5 (0.9)	<0.001
hs-CRP (mean (SD))		4.0 (7.5)	3.6 (6.8)	4.4 (8.1)	<0.001
HT diagnosis (%)		420334242 (36)	191285179 (35)	229049064 (37)	0.078
HT prescriptions (%)		360396752 (86)	165349108 (87)	195047644 (85)	0.142
Now taking HT drugs (%)		310786774 (86)	146248759 (88)	164538016 (84)	<0.001
Controlled HT (ACC/AHA guidelines)	<130/80mmHg	655777691 (56)	317282673 (59)	338495018 (54)	<0.001
Controlled HT (ESC/ESH guidelines)	<140/90mmHg	922957297 (79)	437777267 (81)	485180030 (78)	<0.001
SBP (mean (SD))		125.2 (18.7)	124.0 (18.0)	126.3 (19.2)	<0.001
DBP (mean (SD))		72.3 (12.4)	72.0 (11.9)	72.6 (12.9)	0.009
CAL (mean (SD))		1.3 (1.0)	1.1 (0.8)	1.4 (1.2)	<0.001
PPD (mean (SD))		1.3 (0.5)	1.1 (0.5)	1.4 (0.6)	<0.001
BoP (mean (SD))		8.2 (13.7)	6.0 (10.8)	10.3 (15.7)	<0.001
Missing teeth (mean (SD))		10.3 (9.8)	8.2 (7.7)	12.1 (11.0)	<0.001
Dental implants (%)		36673060 (3)	27643620 (5)	9029440 (2)	<0.001

Smoking (%)		561014309 (48)	234264620 (43)	326749688 (52)	<0.001
Health insurance (%)		999594907 (86)	508196310 (94)	491398598 (79)	<0.001
Dental coverage (%)		235759551 (64)	126635250 (69)	109124302 (59)	<0.001
Last medical visit (%)	<6 Months	2881443 (3)	964911 (4)	1916532 (3)	0.007
	<1 Year	4454213 (5)	1213737 (5)	3240476 (5)	
	<3 Years	52780602 (57)	15736657 (65)	37043944 (55)	
	>3 Years	30749631 (33)	6279369 (26)	24470261 (36)	
	Never	1482533 (2)	201668 (1)	1280866 (2)	
Last dental visit (%)	≤6 Months	542154328 (46)	542154328 (100)	0 (0)	<0.001
	<1Year	168208788 (14)	0 (0)	168208788 (27)	
	<2 Years	130956670 (11)	0 (0)	130956670 (21)	
	<3 Years	77848052 (7)	0 (0)	77848052 (12)	
	<5 Years	78216133 (7)	0 (0)	78216133 (13)	
	>5 Years	158919243 (14)	0 (0)	158919243 (25)	
	Never have been	10403603 (1)	0 (0)	10403603 (2)	
Dental visit reasons (%)	Called for check-up/exam/clean	62554711 (5)	41311934 (8)	21242778 (3)	<0.001
	Check-up/exam/clean	608590295 (53)	348527683 (64)	260062611 (42)	

	Something was wrong/bothering/hurting	324689789 (28)	90755387 (17)	233934401 (38)	
	Treatment of condition discovered at checkup/exam	119453887 (10)	52340116 (10)	67113770 (11)	
Recommendation for cure (%)	Dentist immediately	947572 (0)	163800 (0)	783772 (0)	<0.001
	Dentist within 2 weeks	55638023 (5)	8524713 (2)	47113310 (8)	
	Dentist earliest convenience	467185377 (42)	161497256 (31)	305688121 (52)	
	Continue regular routine care	586561708 (53)	349314894 (67)	237246814 (40)	
NHANES cycles (%)	1999-2000	144340820 (12)	64698893 (12)	79641927 (13)	0.371
	2001-2002	149440732 (13)	69644117 (13)	79796615 (13)	
	2003-2004	152581046 (13)	65720704 (12)	86860342 (14)	
	2011-2012	173255475 (15)	81681083 (15)	91574392 (15)	
	2013-2014	178379565 (15)	88245325 (16)	90134240 (14)	
	2015-2016	185081063 (16)	85484022 (16)	99597041 (16)	
	2017-2018	185059934 (16)	86680183 (16)	98379751 (16)	

PIR: Poverty-Income Ratio; NH: Non-Hispanic; BMI: Body Mass Index; WBC: White Blood Cells; LDL: Low-Density Cholesterol; HbA1C: Glycohemoglobin; CHF: Congestive Heart Failure; CAD: Coronary Artery Disease; CRP: C-Reactive Protein; HS-CRP: High Sensitive C-Reactive Protein; HT: Hypertension; ACC: American College of Cardiology; AHA: American Heart Association; ESC: European Society of Cardiology; ESH: European Society of Hypertension; SBP: Systolic Blood Pressure; DBP: Diastolic Blood Pressure; CAL: Clinical Attachment Loss; PPD: Periodontal Probing Depth; BoP: Bleeding on Probing; NHANES: National Health and Nutrition Examination Survey; SD: standard deviation
