# Supplementary material for: Access to dental care and blood pressure profiles in adults with high socioeconomic status
Source: J Periodontol. 2021 Dec 21;93(7):1060–71. doi: 10.1002/JPER.21-0439 (PMC9542004; doi:10.1002/JPER.21-0439)
Supplement: Supplementary file 4 — Supplementary information [file JPER-93-1060-s001.pdf]

Supplementary Table 2. Overall and age-stratified weighted frequency of medical visits and dental visits among the 27,725 NHANES participants included in the population-based analysis.

Strata	Elapsed time	Medical visit	Dental visit	P value
Overall	≤6 Months	2863736 (3.11)	24396342 (26.50)	<0.001
	<1Year	4454213 (4.84)	13628386 (14.80)	
	<3Years	52581363 (57.12)	22979164 (24.96)	
	3+ Years	30672232 (33.32)	28699802 (31.18)	
	Never	1482533 (1.61)	2350384 (2.55)	
<45	≤6 Months	1322655 (2.47)	12867055 (24.02)	<0.001
	<1Year	2888964 (5.39)	8824027 (16.47)	
	<3Years	31973444 (59.68)	15437816 (28.82)	
	3+ Years	16572218 (30.93)	14803405 (27.63)	
	Never	816979 (1.52)	1641955 (3.06)	
45-65	≤6 Months	1083104 (3.28)	10031293 (30.33)	<0.001
	<1Year	1385924 (4.19)	4412079 (13.34)	
	<3Years	17337933 (52.43)	6653799 (20.12)	
	3+ Years	12660020 (38.28)	11301459 (34.17)	
	Never	604744 (1.83)	673094 (2.04)	
>65	≤6 Months	457978 (8.47)	1497994 (27.70)	0.008
	<1Year	179326 (3.32)	392280 (7.25)	
	<3Years	3269986 (60.46)	887548 (16.41)	
	3+ Years	1439994 (26.63)	2594938 (47.98)	
	Never	60810 (1.12)	35334 (0.65)	
