# Supplementary material for: Access to dental care and blood pressure profiles in adults with high socioeconomic status
Source: J Periodontol. 2021 Dec 21;93(7):1060–71. doi: 10.1002/JPER.21-0439 (PMC9542004; doi:10.1002/JPER.21-0439)
Supplement: Supplementary file 5 — Supplementary information [file JPER-93-1060-s002.pdf]

Supplementary Table 3. Beta estimates and standard errors (SE) for the association of dental visits attendance ≤6 months with selected demographic and clinical features across NHANES campaigns.

	NHANES waves						
	**1990-2000**	**2001-2002**	**2003-2004**	**20011-2012**	**2013-2014**	**2015-2016**	**2017-2018**
Weighted sample	144,340,820	149,440,732	152,581,046	173,255,475	178,379,565	185,081,063	185,059,934
	**β (SE)**	**β (SE)**	**β (SE)**	**β (SE)**	**β (SE)**	**β (SE)**	**β (SE)**
Female	0.194 (0.093)	0.123 (0.049) *	0.175 (0.085)	0.177 (0.072) *	0.135 (0.07)	0.232 (0.079) *	0.2 (0.075) *
45-65 Yrs	0.141 (0.091)	0.352 (0.117) *	0.35 (0.098) **	0.414 (0.095) **	0.41 (0.097) **	0.286 (0.099) *	0.389 (0.154) *
>65 Yrs	-0.202 (0.094)	0.171 (0.077) *	0.151 (0.112)	0.48 (0.122) **	0.534 (0.118) **	0.511 (0.115) **	0.761 (0.164) ***
Underweight	0.308 (0.374)	-0.054 (0.305)	-1.162 (0.501) *	-0.468 (0.34)	-0.621 (0.378)	-0.044 (0.497)	-1.119 (0.585)
Overweight	-0.014 (0.124)	-0.026 (0.098)	-0.032 (0.089)	0.074 (0.137)	-0.168 (0.077)	0.015 (0.12)	0.062 (0.137)
Obese	-0.405 (0.105) **	-0.42 (0.127) **	-0.118 (0.118)	-0.22 (0.146)	-0.551 (0.086) ***	-0.306 (0.098) **	-0.226 (0.09) *
Hispanic	-0.726 (0.11) ***	-0.698 (0.131) ***	-0.79 (0.155) ***	-0.802 (0.145) ***	-0.83 (0.114) ***	-0.879 (0.13) ***	-0.39 (0.117) **
9-11th grade	0.108 (0.181)	0.106 (0.224)	0.73 (0.261) *	0.419 (0.156) *	-0.138 (0.128)	-0.165 (0.225)	0.63 (0.111) ***
College graduate or above	2.022 (0.233) ***	1.804 (0.203) ***	2.165 (0.18) ***	1.882 (0.208) ***	1.658 (0.149) ***	1.632 (0.175) ***	1.695 (0.131) ***
High school graduate/GED	0.808 (0.173) **	0.852 (0.189) **	1.292 (0.253) ***	0.837 (0.161) ***	0.52 (0.176) *	0.378 (0.196)	0.873 (0.144) ***
Some college/AA degree	1.254 (0.175) ***	1.292 (0.161) ***	1.613 (0.197) ***	1.149 (0.166) ***	0.907 (0.187) ***	0.69 (0.12) ***	1.068 (0.153) ***
PIR 130%-350%	0.336 (0.113) *	1.028 (0.138) ***	0.594 (0.083) ***	0.792 (0.114) ***	0.624 (0.134) ***	0.452 (0.133) **	0.583 (0.116) ***
PIR >350%	1.453 (0.186) ***	1.804 (0.162) ***	1.464 (0.137) ***	1.82 (0.115) ***	1.661 (0.12) ***	1.489 (0.118) ***	1.436 (0.117) ***
Diabetes Yes	-0.783 (0.144) ***	-0.449 (0.155) *	-0.425 (0.149) *	-0.137 (0.116)	-0.424 (0.12) **	-0.237 (0.14)	-0.121 (0.117)
HT diagnosis	-0.154 (0.09)	-0.116 (0.091)	-0.09 (0.085)	-0.089 (0.091)	0.01 (0.101)	-0.082 (0.076)	-0.009 (0.086)
SBP (mmHg)	-0.012 (0.002) ***	-0.004 (0.003)	-0.005 (0.002) *	-0.005 (0.002) *	-0.007 (0.003) *	-0.005 (0.002) *	-0.009 (0.003) *
<130/80 mmHg	0.449 (0.087) ***	0.168 (0.072) *	0.133 (0.093)	0.023 (0.097)	-0.007 (0.003) *	0.126 (0.093)	0.225 (0.118)
<140/90 mmHg	0.267 (0.065) **	0.193 (0.096)	0.182 (0.116)	0.102 (0.1)	0.245 (0.139)	0.098 (0.106)	0.248 (0.119)
Smokers	-0.227 (0.103) *	-0.416 (0.078) ***	-0.281 (0.09) **	-0.352 (0.102) **	-0.457 (0.084) ***	-0.447 (0.088) ***	-0.29 (0.109) *
Presence of comorbidities	-0.361 (0.228)	-0.422 (0.199)	0.128 (0.17)	-0.102 (0.172)	-0.414 (0.264)	0.017 (0.15)	-0.232 (0.185)
No insurance	-1.097 (0.107) ***	-1.545 (0.172) ***	-1.369 (0.119) ***	-1.386 (0.15) ***	-1.636 (0.142) ***	-1.556 (0.147) ***	-1.703 (0.214) ***
Called for check-up/exam/clean	0.34 (0.236)	0.585 (0.233) *	0.668 (0.192) **	-0.193 (0.241)	-0.166 (0.409)	0.618 (0.255) *	0.262 (0.231)
Something was wrong/bothering/hurting	-1.142 (0.106) ***	-1.221 (0.075) ***	-1.081 (0.082) ***	-1.518 (0.117) ***	-1.316 (0.117) ***	-1.24 (0.116) ***	-1.156 (0.114) ***
Presence of dental implants	1.092 (0.354) **	0.574 (0.713)	1.089 (0.541)	2.193 (0.358) ***	1.571 (0.223) ***	1.135 (0.208) ***	1.153 (0.25) ***

PIR: Poverty-Income Ratio; Yrs: years; GED: HT: hypertension; SE: standard error
